# Kink in an English Field: The Drinking, Drug use and Sexual Practices of English Festival-Goers Who Engage in Kink

**DOI:** 10.1007/s12119-022-09968-4

**Published:** 2022-04-27

**Authors:** Mark McCormack, Fiona Measham, Maria Measham, Liam Wignall

**Affiliations:** 1 School of Humanities and Social Sciences, University of Roehampton, Roehampton Lane, London, SW15 5PU UK; 2 School of Law and Social Justice, University of Liverpool, Liverpool, UK; 3 University of Cambridge, Cambridge, UK; 4 Department of Psychology, University of Bournemouth, Poole, UK

**Keywords:** Alcohol, BDSM, Drugs, Kink, Leisure, Festivals, Sexual behaviors

## Abstract

Little is known about the other leisure activities of people who engage in kink, including sexual practices and the use of alcohol and other drugs. This article examines the drinking, illicit drug use and sexual practices of people who engage in kink from a novel sample of attendees at an English festival. Of 966 respondents, 64 reported having engaged in kink within the past 12 months. We provide evidence of these respondents’ self-reported demographic characteristics, alcohol and other drug use in their lifetime and within the past 12 months, as well as other sexual practices they engaged in. This study illustrates the value of accessing participants through in situ festival fieldwork to understand kink practices, and helps us move beyond notions of clustered risky activities toward a leisure studies approach to understanding the practices of people who engage in kink.

## Introduction

Kink is a spectrum of sexual and erotic activities outside of normative understandings of sex, undertaken for pleasure (Wignall, 2022). Once viewed predominantly as a sexual perversion through a biomedical lens, kink is better understood as a sexualized form of leisure (e.g., Jackson et al., 2020; Newmahr, 2010). Despite increased academic attention to kink practice and communities, there is little understanding of the other leisure activities and consumption practices of people who engage in kink. Furthermore, the focus on the social and community aspects of kink means a bias exists in research toward understanding kink as it occurs within distinct communities, rather than as it is practiced by individuals in a diverse range of contexts including where leisure activities can take place in more fluid ‘scenes’ and ‘neotribes’ beyond traditional ‘subcultures’ (Blackman, 2014; Wignall, 2022). As such, there is value in adopting a wider range sampling strategies, including using data from people who are part of broader studies not explicitly focused on kink.

In this article, we address these gaps by examining the drug taking and sexual practices of people who engage in kink from a novel sample of 966 English festival-goers. Analyzing survey data from 2016 and 2019, we focus on the respondents who reported having engaged in kink in the past 12 months, and document their demographic characteristics, their other sexual practices and their drinking and drug taking practices. In this way, we are surveying people who engage in kink without necessarily identifying as “kinky” or part of kink communities, providing insight into the leisure-time consumption practices of people who engage in kink from a population broader than much kink research that is focused on people embedded within kink communities while also drawing attention to connections between sexual and drug taking practices in non-stigmatizing ways.

### The Missing Leisure of Kink

A recent trend in research on kink has been to analyze it as a leisure activity, shifting from a pathological framework to one that can critically engage with the benefits and risks of the activity alongside the emotional, economic and psychological labor that individuals invest in kink (Newmahr, 2010; Wignall & McCormack, 2017; Williams et al., 2016). While this shift has provided a fertile framework for understanding diverse communities of kink, the research remains focused on the topic of kink—whether that be as it relates to identities, communities, risk or benefits of the practice. This focus has the consequence of marginalizing the broader lives, relationships and identities of kinky people when it occurs in a paradigmatic way. Such focus can render kink as something separate and distinct from an individual’s broader social life, leisure activities or identity—unintentionally reinscribing the non-normative framing or subcultural aspects of kink.

This is not an issue that is solely the domain of kink, but one that occurs in sexualities and drug research more generally. Research on sex work frequently focuses on the act of exchanging money for sex with the result that the broader lives and identities of sex workers are erased^1^—an issue that has particularly damaging consequences for a group that often experiences harmful legal regulation and police harassment (Kinnell, 2008). Similarly, a prohibitionist legal framework for the control of psychoactive drugs is accompanied by strict police enforcement, enabling classed and racialized stereotyping of drug users that results in discriminatory use of measures such as ‘stop and search,’ leading to over-patrolling (Vomfell & Stewart, 2021) and over-searching (Shiner et al., 2018). This also deters leisure venues and their management from using harm reduction services, meaning that ineffective deterrence and prohibitionist measures are used instead, driving drug use and associated problems further underground, as seen with the US RAVE Act (Ruane, 2015). These troubled relationships with law enforcement have resonance with kink research given the problematic policing and criminalization of consensual kink practice (Khan, 2014). Across these cases, similar patterns of social and legal policing exacerbate problems rather than address them: these groups experience forms of deviance amplification where social problems perceived to be associated with them come not from the groups or their practices but from their labelling as deviant by the dominant culture (Cohen, 1972).

The need to focus on leisure among kinky people may seem apparently less significant in comparison with that of sex workers or people who use drugs because so much recent research on kink already situates these practices within communities, subcultures and scenes, or examines how these practices are connected with social identity and agency (e.g., Newmahr, 2011; Ortmann & Sprott, 2012; Wignall, 2022). Still, understanding the leisure activities of kinky people beyond their kink communities can provide significant insight about their experiences, their social networks, and the broader dynamics of kink in contemporary society, where kink and sex still can be taboo topics that people wish to remain private (Bezreh et al., 2012)—particularly when they intersect with the consumption of alcohol and other drugs (Race, 2009).

While there are many aspects of leisure activities that could be considered in relation to kinky people, in this article we explore use of alcohol and other drugs as well as their other sexual practices, as key forms of leisure activity. These leisure practices also face legal regulation and moral censure (Room, 2005), particularly when consumed by marginalized groups or in specific contexts. Drug taking practices and the resulting state of intoxication are also stigmatized as a form of unearned physical pleasure, rooted in the Victorian rational recreation movement centered on notions of respectable leisure time pursuits and the idea that leisure time should be spent “bettering oneself” (Bailey, 2014; Yeomans, 2014). In this way, the social processes of stigma associated with kink are similarly experienced by people who use drugs, with similar concerns around safety and consent present. The intersections between drug use, kink and sex are complex and have significant implications, and we address this in the following section.

### Connecting Drugs, Sex and Kink

The associations between drugs and kink are complex and, to understand them, we must first consider the intersections of drug taking and sexual practices more broadly. One strand of research on this intersection uses quantitative data to identify associations between drinking, drug use and sexual activity (e.g., Plant & Plant, 1992). Within this paradigm, researchers may focus on specific drugs, such as methamphetamine, GHB/GBL and mephedrone, or on specific risk patterns and negative health outcomes (e.g., Romanelli et al., 2004; Schmidt et al., 2016). For gay and bisexual men, much of the recent focus on the interface of sex and drugs has been on “chemsex,” a stigmatized practice which normally involves a prolonged period of group sex under the influence of drugs; although research contests this dominant framing (Drysdale et al. 2020; Race et al., 2021). This turn towards broader framings is part of a sociological push to recognize the social construction of drugs as gendered and sexualized (e.g., Ettorre, 2004; Measham, 2002).

The sociological turn has highlighted that the perception and regulation of drugs are dependent as much on the *context* of the drug use as the content of the substance (Hartogsohn, 2017; Moore & Measham, 2012; Race, 2009; Zinberg, 1984). This contextual application is particularly sensitive to the ethnicity and social class of the primary consumers, such that illicit substances are more harshly regulated and policed when the predominant consumers are people of color rather than White people (Provine, 2007) or working-class people rather than middle-class people (Palamar et al., 2015). Even as the use of certain psychoactive drugs has become increasingly normalized (Aldridge et al., 2011), the regulation of drugs in the UK has been described as a ratchet where emergent psychoactive substances tend to face increasingly strict legislative controls (Stevens & Measham, 2014); although decriminalization is gaining pace in many countries and particularly for drugs like cannabis (Seddon & Floodgate, 2020).

Race (2009) has pioneered sociological understanding of the social intersections of drugs and sexuality, arguing that the consumption of illicit drugs is a site where moral judgements are made about sexuality, health and pleasure. Despite a dominant cultural narrative of drug regulation being about safety and control, laws and cultural norms construct gay male drug users in biomedical ways that stigmatize their practices and sexual cultures and thus impede access to sexual health services (Race, 2009). His work has spurred a research agenda examining the interactional and discursive meanings of sexual-psychoactive subcultures (e.g., Hakim, 2019; Souleymanov et al., 2019), as well as different approaches to considering the intersections of sex and drugs (McCormack et al., 2021; Moyle et al., 2020).

The use of alcohol and other drugs within a kink context is much less frequently discussed. One reason for this is the stringent focus on safety, consent and control of many kink communities, whether through the most well-known phrase “safe, sane and consensual” (Williams et al. 2014) or other more recent adaptions, such as “risk aware consensual kink” and the “4Cs” framework (consent, communication, caring and caution) (see Wignall, 2020). Within these frameworks, excessive consumption of alcohol or other drugs and the resulting loss of self control could be seen as threats to these rules—most notably of safety, given that some psychoactive drugs may lower inhibitions, change perceptions and affect the ability to fully and freely provide informed consent, putting oneself and other people at risk in such a context.

There are two interconnected issues regarding the consumption of drugs concurrently with kink activities. The first concern is that people could come to harm if people engaging in kink are under the significant influence of alcohol or other drugs. Some activities, such as edgeplay (referring to play where boundaries related to safety and consent are pushed (Newmahr, 2011: p. 147), and sociologically has connections with edge work (Lyng, 2004)), carry higher levels of risk and it is important that individuals can clearly navigate them. Given that some drugs can lower inhibitions and alter the ability to accurately read cues/signals from others, the reasoning goes, individuals may not be able to give informed and/or ongoing consent to participation. However, while ‘extreme drinking’ and drug use to ‘annihilation’ might seem at odds with the stringent focus on safety in kink (Griffin et al., 2009; Martinic & Measham, 2008), the lack of significant overlap between drug taking and kink activities may be because of their similarities rather than their inability to mix. Certain forms of both intoxication and kink can be seen as edgeplay, with participants seeking pleasure through a “controlled loss of control” for themselves and/or for others (Measham, 2002; Newmahr, 2011). For people experienced using drugs, the desired altered state of intoxication is not ‘annihilation’ but achieving a ‘sweet spot’ without tipping over into the excess that risks compromising the psychoactive experience and the health and wellbeing of the participant.

Secondly, stigmatizing tropes of alcohol and other drug use often frame such practices through notions of “addiction” and “harm,” and might be used by mainstream culture in combination with existing stereotypes of kink around “abuse” and “self-harm” to further marginalize kink communities (Holt, 2016). As such, several kink venues have strict rules on the consumption of alcohol and other drugs (Newmahr, 2011; Rubin, 1984) and sobriety is seen as a requisite for engaging in many kink activities. However, this is not a blanket rule and there are cultural differences. Thus, while people who engage in chemsex and “party ‘n’ play” have formed sexual communities, they are mostly distinct from and separate to kink communities and networks.

In this study, we explore the demographics and the sexual and drug taking practices of people who have engaged in kink in the past 12 months. By drawing on a study whose primary focus is exploring the current and historical drug use of English festival-goers, we have a unique data set about this aspect of people who engage in kink in a context where so little information is known about the drug taking practices of people who practice kink, outside of models of harm, addiction and treatment services. As such, we contribute to knowledge of this beyond both the traditional ‘social problems’ approach that more often views these practices through a biomedical lens of risk and harm and the traditional sociological lens that foregrounds subcultural or neotribal connections.

## Methods

### Materials

Data come from a larger ongoing research project known as the annual *English Festival Study* (EFS). The EFS includes annual convenience sample surveys at English summer music festivals from 2010 to 2019 (Turner & Measham, 2019: p. 97), suspended for 2020 because of festival closures due to COVID-19. The EFS provides important data about trends in self-reported alcohol and other drug use, as well as providing information to stakeholders including event management, medical, welfare and support services, with the aim of informing policy and practice such as harm reduction provision. The survey contains questions about respondent demographic characteristics, past and present alcohol and other drug use, and experiences of festival policing and security. It takes 5–10 min to complete, depending on responses. Anonymity is emphasized and details regarding onside support services are provided.

In 2016 and 2019, an additional question was added to the survey at one festival asking whether respondents had engaged in a range of sexual activities in the past 12 months. Respondents were asked: “In the past 12 months, which of the following sexual practices have you engaged in?”, with the options being: anal sex, chemsex, kink, public sex, purchasing sex, selling sex, sex with a friend and sex with a stranger. The list is not intended to be exhaustive of all sexual activities that have potentially become more inclusive in recent years, but a selection that could be indicative of the broader population of possible sexual practices to make asking the question in a short face-to-face survey within a leisure setting practicable. The rationale for the practices included in the survey is provided elsewhere (McCormack et al., 2021). The broader project sought to identify shifts in practices related to non-traditional sexual practices not included in a traditional norm of reproduction-oriented sexual intercourse within a monogamous relationship (McCormack et al., 2021); a norm that was predominant in the mid-twentieth century and has changed significantly since (e.g., Frank & McEneaney, 1999). Evidence shows that people no longer view sexual intercourse as necessarily aimed at reproduction, and oral sex and sex with contraceptives have become normative (Abma & Martinez, 2017; Habel et al., 2018), so we did not ask about these practices.

### Procedure

The survey is administered verbally by a team of trained researchers under the direction of the authors. The researchers record answers (on paper in 2016 and on electronic tablets in 2019), facilitating opportunities to answer queries from respondents, and provide direct assurances of confidentiality (see McCormack et al., 2021). Care was taken in researcher positioning onsite, in order to maximize privacy for respondents when conducting surveys, by being less likely to be overheard by friends, relatives or passing strangers. Evidence shows that face-to-face convenience sample surveys yield similar results as online surveys regarding drug use more generally (Waldron et al., 2020).

Regarding the sex-specific question, this was read out and the options (stated above) were either read verbally or respondents could read the options. If respondents asked what was meant by kink, researchers were trained to give a conversational answer dependent on the rapport with the respondent, with the key point being that kink is another word for BDSM with reference to *50 Shades of Grey*, if necessary. Evaluation of the survey after the event confirmed that this approach was sufficient.

Researchers had access to all areas of the festival and recruited participants from across the whole festival site, and were instructed to recruit participants across diverse demographics, such as age, gender and ethnicity, being mindful to potential selection bias. Anonymity was emphasized to participants and details were provided for onsite support services including medical, welfare, harm reduction and sexual health. Participants were verbally informed that permission for the research was granted by the festival organizer and police, and that ethical approval was obtained from the university where the authors worked at the time. No remuneration was given to participants.

Further details on the history and recent development of the *in-situ* surveys can be found in Turner and Measham (2019: p. 97), and full information about the materials and procedures used by the EFS are provided elsewhere (McCormack et al., 2021).

Descriptive statistics are presented for the entire data set, alongside a breakdown of participants who engaged in kink in the last 12 months. Chi-squared analyses were performed for different variables.

### Participants

Respondents were festival-goers at a popular English festival in 2016 and 2019. The festival is a medium sized family-friendly music festival located in a rural area, with a capacity of approximately 25,000 attendees each year. The entertainment includes popular festival headline acts, live bands, electronic dance music, jazz and comedy. The festival has a solid customer base of attendees and regularly sells out all tickets early each calendar year.

Out of a total sample of 966 responses (n = 530 in 2016, n = 436 in 2019), 6.6% of respondents (n = 64) said they had engaged in kink in the past 12 months (38 in 2016 and 26 in 2019). Table 1 summarizes the demographic characteristics of the respondents who reported having participated in kink alongside the full sample for the two festivals.

**Table 1:** Demographic information

	Kink respondents	Full Sample
Gender	59.4% male (38)	46.5% male (449)
	40.6% female (26)	52.6% female (508)
		0.3% transgender (3)
		0.6% missing (6)
Sexual identity	76.6% straight (49)	89.4% straight (864)
	10.9% Gay/Lesbian (7)	4.8% Gay/Lesbian (46)
	12.5% Bisexual (8)	4.5% Bisexual (43)
		0.7% Other (7)
		0.6% missing (6)
Ethnicity	96.9% White (62)	97.7% White (944)
	1.6% mixed race (1)	0.7% Black (7)
	1.6% missing (1)	0.7% Asian (7)
		0.4% Mixed (4)
		0.2% other (2)
		0.2% missing (2)
Employment status	60.9% Employed (39)	63.9% Employed (617)
	34.3% in education (22)	31.1% in education (300)
	1.6% other (1)	4.0% other (39)
	1.6% missing (1)	1.0% missing (10)
Mean age	26.2	28
Age SD	7.8	10.4
Median age	24.0	25

There is little research on prevalence of kink practices in the general population: a 2008 representative survey of Americans found that 1.8% reported BDSM in the past 12 months (Richters et al., 2008). By comparison, the EFS found that prevalence of self-reported kink, along with self-reported drug use, was considerably higher in this sample of festival-goers than in the general population. This may be due to the non-representative nature of the characteristics of those attending festivals: for example, festival-goers have higher rates of consumption of alcohol and other drugs than the general population, as well as being on average over ten years younger than the general adult population.

The majority of participants who said they engaged in kink were male (59.4%) and heterosexual (76%). The mean age of kinksters was 26.2 years, and the great majority self-identified as White (96.9%), similar to the full sample and the demographics of the region in which the festival occurs.

A little under two thirds (60.9%) of kink respondents were in employment (somewhat lower than the full sample), and just over one third (34.3%) were in education (somewhat higher than the full sample), suggesting kink respondents are slightly more likely to be students. This also corresponds with the slightly younger age range of kink respondents compared with the general festival sample.

## Results

In the following section, we examine for associations between engagement in kink and demographic characteristics; sexual practices; and alcohol and other drug use.

### Gender and Sexuality

A Chi-squared test of association was performed, and a significant relationship was found between self-reported engagement in kink in the last 12 months and sexual orientation: *X*^2^ (1, = 955) = 14.646, *p* < 0.001. Cramer’s V was 0.124, showing a weak effect size. Specifically, people who engage in kink are more likely to be Lesbian, Gay or Bisexual (LGB) than heterosexual compared to the full sample.

A Chi-squared test of association was performed, and a significant relationship was found between engagement in kink in the last 12 months and gender: *X*^2^ (1, = 955) = 4.279, *p* < 0.05. Cramer’s V was 0.067, showing a weak effect size. Specifically, people who engage in kink are more likely to be male than female compared to the full sample.

### The Sexual Practices of People Who Engage in Kink

The self-reported prevalence of each sexual activity is listed in Table 2. In this sample, kink was less prevalent than several other sexual practices although notably more prevalent than chemsex and monetary exchange for sex (purchasing and selling sex were combined given small number of positive responses).

**Table 2:** Sexual activity in the past 12 months

Activity in past 12 months	n = 966	% of the sample
Sex with a friend	268	27.7
Sex with a stranger	181	18.7
Anal sex	107	11.1
Public sex	103	10.7
Kink	64	6.6
Chemsex	18	1.9
Monetary exchange for sex	15	1.6

We performed several Chi-squared tests of association to explore whether having practiced kink in the past 12 months was associated with the other liberal sexual practices. All the liberal sexual practices were associated with engaging in kink in the last 12 months (see Table 3). Anal sex and public sex had medium effect sizes, while chemsex, sex with a stranger, and sex with a friend had small effect sizes.

**Table 3:** Chi squared tests of association for kink and other sexual practices

		Kink in the last 12 months		Chi square tests of association	
		No	Yes	X^2^	V
Anal sex	No	832	25	172.553*	0.423
	Yes	68	39		
Chemsex	No	889	57	30.778*	0.179
	Yes	11	7		
Public sex	No	830	31	120.034*	0.353
	Yes	70	33		
Sex with a friend	No	663	33	14.545*	0.123
	Yes	237	31		
Sex with a stranger	No	747	36	28.035*	0.171
	Yes	153	28		

* = significant at *p* < 0.001.

### Alcohol and Other Drug Use by Kinksters

We were also interested in self-reported use of alcohol and other drugs by those who engaged in kink. Respondents were asked to assess their usual frequency of alcohol consumption and those reporting engaging in kink also reported more frequent drinking. Table 4 shows the rates of drinking, with a greater preponderance of people who had engaged in kink reporting more frequent alcohol consumption than the full sample.

**Table 4:** Self-reported average drinking for kinksters and the full sample

Drinking	Kinksters	Full sample
Every day	4.7% (3)	2.6% (25)
Most days a week	23.4% (15)	11.7% (113)
2/3 times a week	35.9% (23)	35.8% (346)
Once a week	21.9% (14)	27.5% (266)
Once a fortnight	12.5% (8)	9.8% (95)
Once a month	0	6.2% (60)
Less than once a month	1.6% (1)	1.8% (17)
Never drink	0	4.1% (40)
Missing	0	0.4% (4)

In addition to alcohol consumption, we performed several Chi-squared tests of association to explore whether having practiced kink in the last 12 months was associated with self-reported use of a series of illicit drugs within the last 12 months. The six drugs we selected are commonly associated with leisure time partying, indicative of a broader normalization of recreational drug use in British culture (Aldridge et al., 2011): cannabis, cocaine, ketamine, MDMA, LSD and ‘poppers’ (alkyl nitrite in the UK context).

Of the illicit drugs, ketamine (*p* < 0.05), LSD (*p* < 0.05), MDMA (*p* < 0.005) and poppers (*p* < 0.005) were significantly associated with kink (see Table 5 for details of the Chi-squared tests of association), although all with small effect sizes. Cannabis and cocaine were not associated with kink.

**Table 5:** Chi square tests of association for kink and drug use

		Kink in last 12 months		Chi square tests of association	
		No	Yes	X^2^	V
Cannabis in last 12 months	No	543	31	3.510	0.060
	Yes	357	33		
Cocaine in the last 12 months	No	660	40	3.526	0.060
	Yes	240	24		
MDMA pills in the last 12 months	No	733	42	9.487**	0.099
	Yes	167	22		
LSD in the last 12 months	No	858	56	7.455*	0.088
	Yes	42	8		
Poppers in the last 12 months	No	832	52	9.839*	0.101
	Yes	68	12		
Ketamine in the last 12 months	No	779	48	6.544*	0.082
	Yes	121	16		
Polysubstance in the 12 months	No	628	37	3.998*	0.064
	Yes	272	27		

* = significant at *p* < 0.05.

* = significant at *p* < 0.005.

We also explored whether consuming two or more illegal drugs and alcohol within the last 12 months (termed concurrent polysubstance use^2^) was associated with engaging in kink in the same time period: this was also significant (*p* < 0.05), although also with a small effect size.

## Discussion

This study has presented data on attendees at an English music festival who reported having engaged in kink practices in the prior 12 months. From a sample of 966 respondents, one in fifteen (6.6%) had engaged in kink in the past 12 months. This group was more likely to identify as belonging to a sexual minority than the broader sample and also more likely to identify as male rather than female compared to the broader sample.

One significant finding of this research is the lack of palpable real-world difference in self-reported use of alcohol and other drugs between festival-goers who engaged in kink and those who did not. Our statistical analysis showed that while connections exist, the effect sizes mean they are unlikely to reflect a palpable difference in real life practices. This challenges the traditional view of clustered risk-taking behaviors (e.g., Plant & Plant, 1992) and supports the broader argument that positions both kink and recreational drug use as increasingly normalized leisure activities that are not disproportionately or inevitably associated with problematic, dependent or harmful practices (e.g., Aldridge et al., 2011; McCormack et al., 2021; Moyle et al., 2020). In showing similar drug taking practices to non-kinksters, it provides further evidence to support the idea of both kink and drug use being leisure activities rather than problematic behaviors.

Equally notable was the significant association with other non-traditional sexual practices. People who participated in kink in the past 12 months were significantly more likely to have engaged in anal sex, public sex, sex with a friend, sex with a stranger and chemsex than the full sample; and a medium effect size was reported for anal sex and public sex. This suggests that kink is associated with other non-traditional sexual practices (McCormack et al., 2021; Twenge et al., 2015) and that people who engage in kink might be more likely to have more liberal sexual practices than the general population, or are less constrained by the prevailing norms of sexuality that persist even as more diverse sexual practices become increasingly accepted (McCormack, 2012; McCormack et al., 2021). It is also feasible that the people who reported engaging in kink might score higher on measures of sensation-seeking behaviors, having a greater interest in novel and interesting experiences than others, or they might have more unrestricted sociosexuality (permissive attitudes toward casual sex) (see Vrangalova & Ong, 2014). Future research could investigate whether and how sensation-seeking and sociosexuality may impact on intersections of sexual practice and drug-use and how the impact on notions of risk-taking in these contexts (see Cross et al., 2011).

Our quantitative survey approach provides an important strand to interdisciplinary research on BDSM and kink, where there is a preponderance of qualitative research, particularly in cultural studies. First, drug research tends to interdisciplinarity because it is subject-led rather than discipline-led, spanning chemistry, criminology, cultural studies, public health, psychology and sociology among others; then, by drawing on research and concepts in drug research and bringing them into conversation with sexualities research (and kink research specifically), we make connections and arguments that bridge the disciplines Furthermore, our quantitative approach is at odds with the predominant strand of qualitative methodologies used in research on kink. By examining for associations between kink, sexual practices and alcohol and other drug use as forms of leisure activity, we provide a methodological approach that is often marginalized in research on kink and sexualities research more broadly (Ghaziani & Brim, 2019).

This approach also connects with a second issue: the focus in recent research on how kink is located within subcultures and how these form meaningful sexual communities (e.g., Moskowitz & Roloff, 2007; Rubin, 1991, 2002; Sisson, 2007). While the focus on community and the social aspects of kink are important, it also means that there is a bias in research toward understanding kink practice as it occurs within communities, whether that be in person or online via websites such as FetLife. Wignall (2022) illuminates the kink practices of the “non-community participant” (a person who engages in kink but does not belong to kink communities) and documents differences from kink community members. Similarly, Measham (2019) found community members more likely than non-community participants to report engaging in certain kink activities that are perceived to carry more risk. Yet, recruiting participants for kink research from the general population remains rare (see Holvoet et al., 2017 and Richters et al., 2008 as exceptions), furthering the bias toward hearing narratives of people who are members of kink communities. This is an issue that is found in sexualities research beyond kink, for example with evidence about the impact of pornography consumption being deeply influenced by the sample of the study (see McCormack & Wignall, 2017). While we did not ask participants whether they had strong ties to kink communities, which means that they might also be part of a kink community, the novel sampling strategy is still significant in likely attracting a broader set of people who engage in kink and may or may not be kink community members (see also McCormack, 2014 regarding the value of diverse sampling strategies).

Our finding of one in fifteen respondents having engaged in kink in the past 12 months is higher than some previous studies (e.g., Richters et al., 2008), but it is lower than might be expected, particularly given the notion that diverse sexual practices have become increasingly normalized in the UK. One potential issue is that research has found that reporting rates change depending on whether you ask about self-identification as a kink practitioner versus engaging in specific activities (Coppens et al., 2019). Furthermore, while we asked about activities rather than self-identification, and the researchers were trained regarding how to explain what kink included, it is possible reported rates of kink would be higher if we had listed several practices as examples. This is not possible within the bounds of a short questionnaire in a leisure context where time is at a premium. Relatedly, if kink remains stigmatized for some people, it is quite possible that we recorded false negatives as people decided not to disclose this interest. However, the same would also apply to other activities such as chemsex and exchange of sex for money and the differing response rates for these activities suggest this has not been a prohibitive factor (see Table 2).

Considering the leisure activities of kinky people beyond their particular kink community opens up several possible areas of study. One might consider whether kinky people compartmentalize their kink activities and communities as with other aspects of their lives (Jaspal, Lopes and Wignall, 2020), or whether these are connected to other aspects of their lives. Similarly, it would be interesting to understand better how people who engage in kink participate in heterosexual and queer sexual and leisure spaces more broadly, whether LGB kinksters spend time in “gayborhoods” and whether the sexual conservatism of lesbian and gay politics of the 1970s persists (Rubin, 1981). Future research could also explore the extent to which people who engage in kink may seek sobriety during sexual edgeplay whilst not excluding the use of intoxicants or engagement in psychoactive edgeplay from their lives completely, perhaps suggesting a compartmentalization of physical pleasures. By investigating further the intersections between leisure sex and recreational drug use identified in our research, such studies can also help advance theoretical debates around normalization, detraditionalization and broader shifts in social identities, leisure practices and late modern societies.

## Endnotes

1. We are grateful to Victoria Holt for drawing our attention to this issue in sex work and related fields.

2. While different definitions of polysubstance use exist, we focus on concurrent rather than simultaneous polysubstance use as it is better for exploring trends in drug-taking careers and associated behaviors across a broader time frame.
